# Primary CSF-lymphatic fistula: a previously unknown cause of spontaneous intracranial hypotension

**DOI:** 10.1007/s00415-024-12598-5

**Published:** 2024-08-06

**Authors:** Niklas Lützen, Katharina Wolf, Amir El Rahal, Florian Volz, Theo Demerath, Charlotte Zander, Claus Christian Pieper, Marius Schwabenland, Horst Urbach, Jürgen Beck

**Affiliations:** 1 Department of Neuroradiology, Medical Center-University of Freiburg, Faculty of Medicine, University of Freiburg, Breisacher Str. 64, 79106 Freiburg, Germany; 2 Department of Neurosurgery, Medical Center-University of Freiburg, Faculty of Medicine, University of Freiburg, Freiburg, Germany; 3 Division for Minimally-Invasive Lymph Vessel Therapy, Department of Diagnostic and Interventional Radiology, University of Bonn, Bonn, Germany; 4 Institute of Neuropathology, Medical Center-University of Freiburg, Faculty of Medicine, University of Freiburg, Freiburg, Germany

Dear Sirs,

Spontaneous intracranial hypotension (SIH) is a serious disease that has gained increasing clinical and scientific attention in recent years. It is characterized by orthostatic headache, but may be associated with various other symptoms that can hamper the clinical diagnosis [1]. A spectrum of complications including subdural hematomas, infratentorial siderosis and brain sagging with dementia or coma as the most severe presentation is also known [1]. The incidence of SIH is estimated at 5 cases per 100,000 population, but there is evidence of underdiagnosis.

The cause of SIH is a spinal CSF loss. A ventral dural tear, often associated with a bone spur, and a lateral dural tear at the nerve root sleeve have been established as type 1 and 2 leaks, respectively. A recent breakthrough has been the discovery of CSF-venous fistula (CVF, type 3) as a major cause of SIH resulting in new diagnostic and therapeutic strategies. First described in 2014 by Schievink et al., it was initially detected in 2.5% of SIH patients [2]. A CVF can be challenging to diagnose, but with further advances in imaging techniques, it is currently made in > 25% of cases [3]. Treatments include surgical ligation, percutaneous CT-guided fibrin patch [4] and endovascular transvenous glue embolization [5].

We report the first case of a primary CSF-lymphatic fistula in a 30-year-old woman with SIH. A sole or primary CSF-lymphatic fistula that is not secondary to vascular malformation has not yet been described and can be considered a novel condition with clinical implications. The CSF-lymphatic fistula in this case was detected by myelography and MR lymphangiography. After surgical ligation, SIH signs on MRI resolved and patient recovered completely from orthostatic headache.

A 30-year-old female patient experienced a sudden onset of positional headache. As the intensity of her headache increased, it was no longer controllable with analgesics and caffeine, and became unbearable when standing (intensity of 10/10 on a numeric rating scale). She was referred to a local hospital with a suspected subarachnoid hemorrhage. MRI of the head revealed typical signs of SIH (Fig. 1A, B; 1st MRI). However, MRI of the spine showed no epidural fluid collection. After treatment with caffeine (3 × 200 mg/day) and bedrest, symptoms vanished only for some days, but returned unchanged after cessation. She was unable to work for 6 months from this date.

**Fig. 1:**
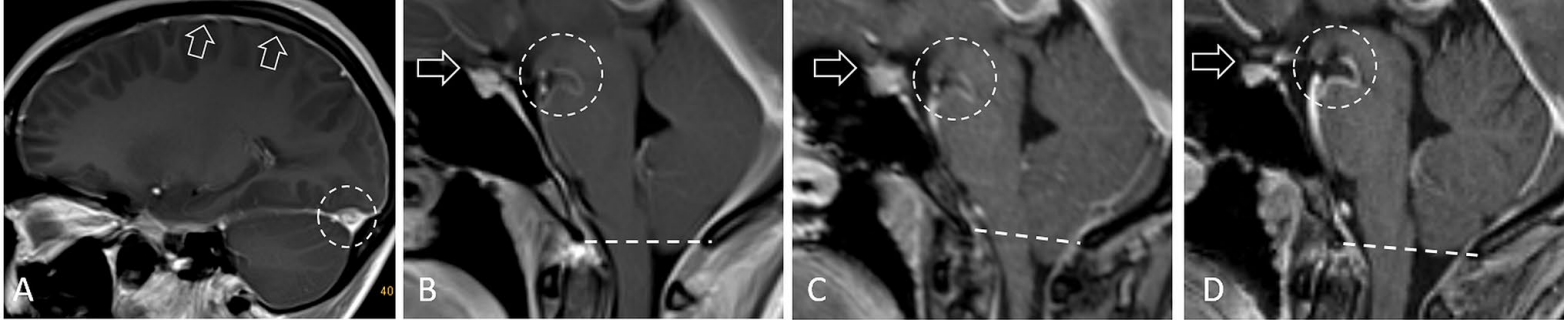
Imaging signs on MRI of the head. First MRI (see also timeline, supplementary file 1) showing typical intracranial signs of spontaneous intracranial hypotension (SIH) with pachymeningeal enhancement (open arrows in **A**) and engorgement of transverse sinus (dashed-line circle in **A**) as well as narrowing of the suprasellar (open arrow in B) and mamillopontine distance (dashed-line circle in **B**). Sagging of the cerebellar tonsil is only slightly visible (dashed-line in **B**). Second MRI is unchanged (not shown). At third MRI 11 months after onset of symptoms, pachymeningeal enhancement and venous engorgement disappeared (not shown), but sagging of the cerebellar tonsil increases (dashed-line in **C**). At the fourth MRI 3 months after surgical ligation of the CSF-lymphatic fistula, suprasellar and mamillopontine distances, and tonsil herniation normalized (**D**)

In the further course, digital subtraction myelography (DSM) and CT myelography (CTM) were performed at an external university hospital, where a CSF leak was suspected due to a dural tear at the level of L4/5 on the left. Two CT-guided lumbar epidural blood patches, strict bedrest and pain therapy only temporarily relieved symptoms. Orthostatic headache was accompanied by various symptoms, such as neck pain, dizziness, nausea, vomiting and cognitive impairment, over the course of 12 months (the patient’s medical history is presented as a timeline in supplementary file 1).

On admission at our hospital, the patient reported positional head and neck pain, which was still at an average intensity level of 4/10 on a numeric rating scale, rarely orthostatic dizziness, while she reported a marked decrease in cognitive performance. She was now capable of staying upright for 4 h a day. The clinical examination revealed no neurological deficits but borderline impaired attention and concentration (Montreal Cognitive Assessment Score 27/30 points). No abnormal laboratory parameters were found.

On MRI of the head, pachymeningeal enhancement and subdural hygromas had disappeared compared to previous imaging eight month ago. Instead, tonsil herniation had increased (Fig. 1C, 3rd MRI) and a new syringomyelia of the cervical spine became visible (online video, supplementary file 2), that was asymptomatic. An epidural CSF collection, as would be expected in case of a spinal dural tear, was still not visible. Consequently, we suspected a CSF-venous fistula.

A left-sided DSM was performed after lumbar puncture at the L5/S1 level. Opening pressure was 0 cm of H_2_O. DSM at the angiography suite showed a tubular paravertebral contrast egress at the L4/5 level left (Fig. 2A and online video, supplementary file 2). A recently introduced ultrahigh-resolution cone-beam CT (UHR-CBCT) myelography [6] providing a spatial resolution of 0.14 mm was supplemented on the same level by rotating one X-ray tube. This revealed contrast medium drainage branching ventrally into the prevertebral region and laterally into the left abdominal trunk, both deviating from the expected anatomy and morphology of paravertebral veins, but compatible with lymphatic vessels (Fig. 2B, C).

**Fig. 2:**
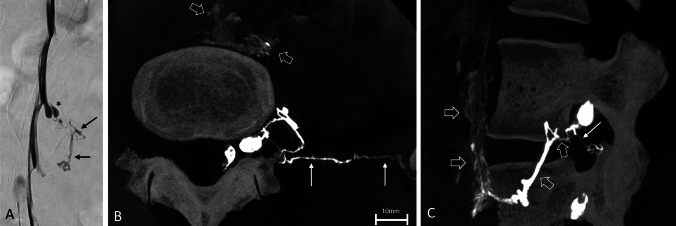
CSF-lymphatic fistula demonstrated by 2D and 3D myelography techniques. Digital subtraction myelography (DSM) in left lateral decubitus position shows an egress of contrast medium, exiting from a small nerve root diverticulum (asterisks in **A**) at the level L4/5 on the left into irregular shaped para-spinal vessels (black arrows in **A**). Ultrahigh-resolution cone-beam CT (UHR-CBCT) myelography depicts a CSF-lymphatic fistula (CLF) with two main drainage pathways: a ventral drainage along paravertebral and retroperitoneal lymphatic vessels (open white arrows in **B** and **C**) and a lateral drainage into the left abdominal trunk (solid white arrows in **B** and **C**)

A left-sided CTM of the entire spine was supplemented at the CT scanner, as the drainage of the CSF-fistula was not fully covered by DSM or UHR-CBCT. Here, contrast medium drained further into the para-aortic lymphatics, thoracic duct and left lymphatic-venous junction (Fig. 3B, C), and on the other hand, into lymphatic vessels of the left abdominal trunk up to intercostal lymphatics (Fig. 3D).

**Fig. 3:**
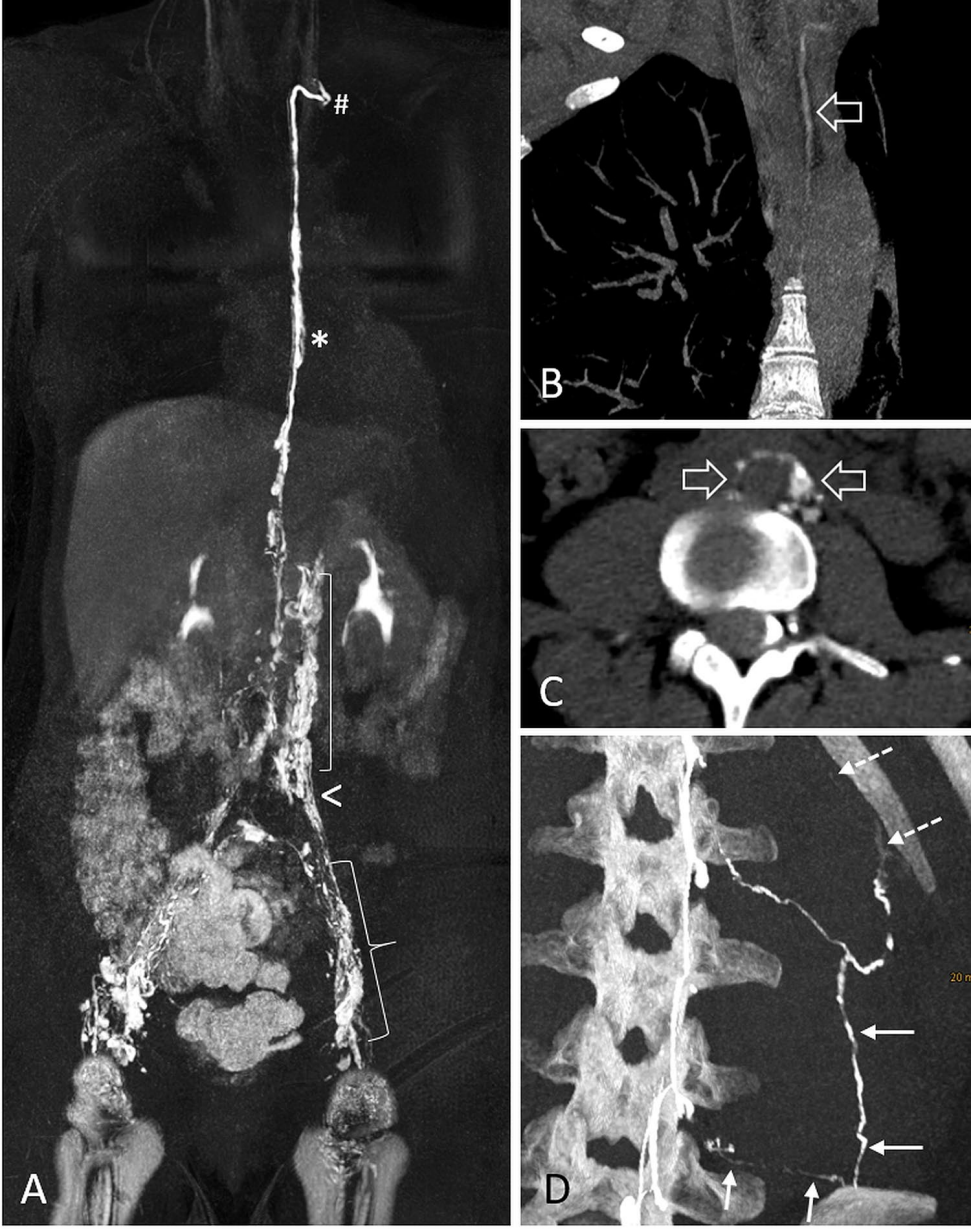
Physiological lymphatic pathways and drainage of the CSF-lymphatic fistula. MR lymphangiography (MRL) visualizing lymphatic drainage pathways from inguinal lymph nodes into iliac (curly bracket in **A**) and retroperitoneal lymphatics (square bracket in **A**), further into the lymphatic duct (asterisks in **A**) and finally into the left lymphatic-venous junction (rhomb in **A**), excluding an underlying lymphatic malformation. White arrowhead in A indicates the level at which the CLF entering the retroperitoneal lymphatic vessels. CT myelography (CTM) also showing contrast drainage into the left thoracic duct (open arrow in **B**) and para-aortic lymphatic chain outlining the aorta (open arrows in **C**). On the other hand, lateral drainage via the left abdominal trunk into lumbar paravertebral (solid arrows in **D**) and intercostal lymphatics is visible (dashed arrows in **D**)

MR lymphangiography (MRL) was performed in a specialized external center showing neither abnormalities nor lymphatic congestion or lymphatic malformation (Fig. 3A). Overlay of the CSF-lymphatic fistula (CLF) using 3D-MRL and 3D-UHR-CBCT showed a common drainage pathway into the para-aortic lymphatics, confirming a primary CSF-lymphatic fistula (online video, supplementary file 2).

12 months after onset of symptoms, the patient underwent minimally invasive surgery at the L4/5 level on the left: hypertrophic vessels in the neuroforamen and tiny vascular structures in the dura around the nerve root sleeve were identified as pathological findings. However, a reliable intraoperative differentiation between lymphatic and blood vessels was not possible. The vessels could be coagulated and draped with resorbable hemostatic galantine, and the dura was augmented with fibrin glue as an adhesive material. A tiny specimen of the fistula could be obtained for histological assessment. Circularly arranged connective tissue with a lumen in the center could be observed. The lumen showed a single-layered lining. These cells were positive in the immunohistochemical reactions for CD31 as well as vWF, which identified them as endothelial cells. Taken together, the histopathological examinations confirmed the presence of a luminal structure with endothelial lining and that this CSF-lymphatic fistula could successfully be removed during surgery. After surgery, the patient developed rebound intracranial hypertension, which responded well to acetazolamide (500 mg/day), and local back pain, which gradually improved. All clinical symptoms resolved within a few days, and at the 3-month follow-up, MRI signs of the head (Fig. 1D, 4th MRI) as well as the spinal syringomyelia had completely disappeared (supplementary video).

We present a case of a sole or primary CSF-lymphatic fistula (CLF) as a novel cause of spontaneous intracranial hypotension confirmed by modern myelography and MR lymphangiography techniques. Any underlying lymphatic abnormalities have been ruled out.

CSF-lymphatic fistulas causing SIH have only been reported in a few cases and exclusively in association with a lymphatic or venolymphatic spinal malformation [7, 8], in particular in Gorham–Stout disease [9, 10], Klippel–Trenaunay syndrome [11] and kaposiform lymphangiomatosis [12]. In these cases, the development of the CLF was attributed to invasive vascular proliferation.

The pathomechanism of the CLF in our case is unclear. CVFs are hypothesized to result from the rupture of a spinal arachnoid granulation that is naturally involved in the venous resorption of CSF. These are known to occur predominantly in the thoracic spine in humans and therefore matching the predilection site of CVFs that is between the T7 and T12 level [13]. In contrast, according to studies on mammals, natural CSF resorption into the para-spinal lymphatic system predominantly takes place along the lumbosacral nerve roots (in particular along dorsal nerve roots) [14, 15], suggesting that CLFs, as in our case at the L4/5 level, may be more likely to be expected in the lower spine.

The differentiation between the two conditions, CVF and CLF, was difficult in this case but could be important in future cases, as modified diagnostic and therapeutic strategies may be required for CLF. A currently widely used treatment option for CVF is transvenous embolization, which would naturally be ineffective for CLF without dedicated access to the lymphatic fistula site. In analogy to established catheter-guided translymphatic embolizations [16], new treatment approaches may be conceivable for CLF: an ultrasound-guided puncture of the cisterna chyli/thoracic duct or alternatively transvenous probing to the left lymphatic–venous junction with subsequent retrograde access to the thoracic duct and finally to the CLF [16]. A further possible treatment option could be a percutaneous CT-guided fibrin patch, as is already used for CVF, however, its efficacy for CLF would first have to be tested [17]. Surgical ligation, as in our case, should be readily available for treatment of CLF.

The lymphatic drainage pathways of the spine are key to understanding CLF and can be briefly described as follows: a network of lymphatic vessels/capillaries wraps around the spinal meninges and drains at each spinal segment dorsally to the ligamentum flavum and ventrally via the neuroforamina, finally reaching the left venous angle via cervical, mediastinal or, for example, retroperitoneal lymph node stations the cisterna chili and the thoracic duct [15]. Whereas if the lymph comes from the right side of the neck or right hemithorax, it drains via right-sided lymph node stations into the right lymphatic duct and finally into the right venous angle. However, it should be noted that the lymphatic system is characterized by a high degree of complexity and variability [16]. The use of MR lymphangiography can help identify the lymphatic draining pathways at myelography and exclude or verify lymphatic malformations.

Imaging of the primary CLF was characterized by high-contrast irregular vessels with frequently changing vessel caliber (caused by multiple lymphatic valves, Fig. 2) and tiny linear lymphatic chains along the para-aortal lymphatics that outline the aorta (Figs. 2, 3C). Moreover, contrasting of the cisterna chyli and thoracic duct (or right lymphatic duct) may be expected on imaging (Fig. 3B). This is different from para-spinal veins and the azygos system, which have a more regular vessel shape and a relatively ordered anatomy. High-resolution 3D myelography techniques of the lower spine visualizing the anatomy in detail, in combination with MR lymphangiography, may be decisive for future diagnoses of CLF. It should also be considered that primary CLFs may be underdiagnosed. Patients who have been diagnosed with CVF and have not responded to transvenous treatment, for example, may have an underlying undiagnosed primary CLF and could benefit from re-evaluation of the draining pathway.

In conclusion, a primary CSF-lymphatic fistula unrelated to vascular malformation is a potential cause of SIH that should be recognized on imaging to facilitate appropriate treatment.

## Supplementary Information

Below is the link to the electronic supplementary material.Below is the link to the electronic supplementary material.

Timeline of patient’s medical history with diagnoses, treatment, symptoms and MR imaging Supplementary file1 (DOCX 993 kb)

Video 1: Digital subtraction myelography (DSM) showing the primary CSF-lymphatic fistula in anterior-posterior projection; Video 2: Ultrahigh-resolution cone-beam CT (UHR-CBCT) myelography showing the primary CSF-lymphatic fistula as 3D pirouette (180°); Video 3: Fusion imaging from ultra-high-resolution cone-beam CT (UHR-CBCT, orange) covering 11 cm of the spine and MR lymphangiography (white) showing a common lymphatic drainage (as a 3D pirouette); Video 4: Superimposition of pre- and postoperative MRI images of the head and cervical spine, showing the disappearance of cranial SIH signs and spinal myelopathy Supplementary file2 (MP4 20040 kb)

## Data Availability

Anonymized data not published within this article will be made available by reasonable request from any qualified investigator.

## References

[1] Dobrocky T, Nicholson P, Häni L et al (2022) Spontaneous intracranial hypotension: searching for the CSF leak. Lancet Neurol 21:369–380. 10.1016/S1474-4422(21)00423-3 35227413 10.1016/S1474-4422(21)00423-3

[2] Schievink WI, Moser FG, Maya MM (2014) CSF-venous fistula in spontaneous intracranial hypotension. Neurology 83:472–473. 10.1212/WNL.0000000000000639 24951475 10.1212/WNL.0000000000000639

[3] Roytman M, Salama G, Robbins MS, Chazen JL (2021) CSF-venous fistula. Curr Pain Headache Rep 25:5. 10.1007/s11916-020-00921-4 33475890 10.1007/s11916-020-00921-4

[4] Kranz PG, Amrhein TJ, Gray L (2017) CSF venous fistulas in spontaneous intracranial hypotension: imaging characteristics on dynamic and CT myelography. AJR Am J Roentgenol 209:1360–1366. 10.2214/AJR.17.18351 29023155 10.2214/AJR.17.18351

[5] Brinjikji W, Savastano LE, Atkinson JLD et al (2021) A novel endovascular therapy for CSF hypotension secondary to CSF-venous fistulas. AJNR Am J Neuroradiol 42:882–887. 10.3174/ajnr.A7014 33541895 10.3174/ajnr.A7014 PMC8115355

[6] Lützen N, Beck J, Urbach H (2023) Cerebrospinal fluid venous fistula imaging with ultrahigh-resolution cone-beam computed tomography. JAMA Neurol 80:870–871. 10.1001/jamaneurol.2023.1640 37306975 10.1001/jamaneurol.2023.1640

[7] Schievink WI, Maya MM, Moser FG et al (2019) Spontaneous spinal CSF-venous fistulas associated with venous/venolymphatic vascular malformations: report of 3 cases. J Neurosurg Spine 32:305–310. 10.3171/2019.8.SPINE19716 31675703 10.3171/2019.8.SPINE19716

[8] Schievink WI, Maya MM, Borst AJ (2023) Adolescent headache due to congenital pelvic/sacral vascular malformation. Ann Neurol 93:1214–1215. 10.1002/ana.26651 36995356 10.1002/ana.26651

[9] Suero Molina EJ, Niederstadt T, Ruland V et al (2014) Cerebrospinal fluid leakage in Gorham-Stout disease due to dura mater involvement after progression of an osteolytic lesion in the thoracic spine. J Neurosurg Spine 21:956–960. 10.3171/2014.8.SPINE131064 25325172 10.3171/2014.8.SPINE131064

[10] Adler F, Gupta N, Hess CP et al (2011) Intraosseous CSF fistula in a patient with Gorham disease resulting in intracranial hypotension. AJNR Am J Neuroradiol 32:E198-200. 10.3174/ajnr.A2413 21659480 10.3174/ajnr.A2413 PMC7964379

[11] Madhavan AA, Kim DK, Carr CM et al (2020) Association between Klippel-trenaunay syndrome and spontaneous intracranial hypotension: a report of 4 patients. World Neurosurg 138:398–403. 10.1016/j.wneu.2020.03.148 32247792 10.1016/j.wneu.2020.03.148

[12] Soderlund KA, Mamlouk MD, Shah VN et al (2021) Cerebrospinal fluid-lymphatic fistula causing spontaneous intracranial hypotension in a child with kaposiform lymphangiomatosis. Pediatr Radiol 51:2093–2097. 10.1007/s00247-021-05132-6 34286352 10.1007/s00247-021-05132-6 PMC8294238

[13] Kranz PG, Gray L, Malinzak MD et al (2021) CSF-venous fistulas: anatomy and diagnostic imaging. AJR Am J Roentgenol 217:1418–1429. 10.2214/AJR.21.26182 34191547 10.2214/AJR.21.26182

[14] Proulx ST (2021) Cerebrospinal fluid outflow: a review of the historical and contemporary evidence for arachnoid villi, perineural routes, and dural lymphatics. Cell Mol Life Sci 78:2429–2457. 10.1007/s00018-020-03706-5 33427948 10.1007/s00018-020-03706-5 PMC8004496

[15] Gonuguntla S, Herz J (2023) Unraveling the lymphatic system in the spinal cord meninges: a critical element in protecting the central nervous system. Cell Mol Life Sci 80:366. 10.1007/s00018-023-05013-1 37985518 10.1007/s00018-023-05013-1 PMC11072229

[16] Pieper CC (2023) Back to the future II-A comprehensive update on the rapidly evolving field of lymphatic imaging and interventions. Investig Radiol 58:610–640. 10.1097/RLI.0000000000000966 37058335 10.1097/RLI.0000000000000966

[17] Mamlouk MD, Shen PY, Sedrak MF, Dillon WP (2021) CT-guided fibrin glue occlusion of cerebrospinal fluid-venous fistulas. Radiology 299:409–418. 10.1148/radiol.2021204231 33650903 10.1148/radiol.2021204231

