# Supplementary figures and images for: Primary CSF-lymphatic fistula: a previously unknown cause of spontaneous intracranial hypotension
Source: J Neurol. 2024 Aug 6;271(10):7016–20. doi: 10.1007/s00415-024-12598-5 (PMC11447140; doi:10.1007/s00415-024-12598-5)

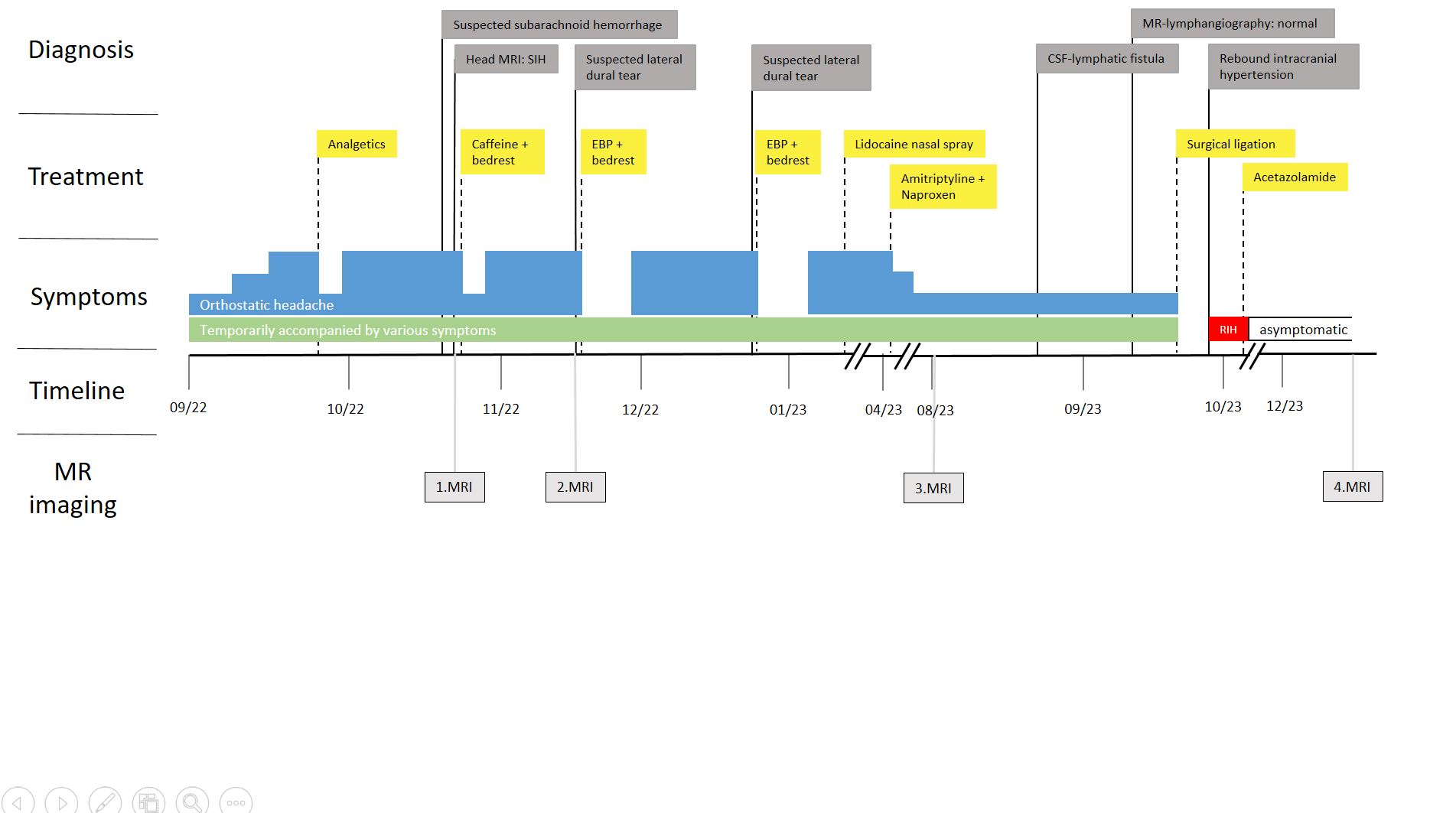

Supplement: Supplementary file 1 — Timeline of patient’s medical history with diagnoses, treatment, symptoms and MR imaging Supplementary file1 (DOCX 993 kb) [file 415_2024_12598_MOESM1_ESM.jpg]
